# The mediating role of self-esteem in the link between attachment relationships and stress among Vietnamese nursing students: a cross-sectional survey

**DOI:** 10.1016/j.ijnsa.2026.100500

**Published:** 2026-01-31

**Authors:** Quynh Brooke Ho, Huong Thi Lan Tran, Dung Thi Thuy Nguyen, Thuan Thi Tran, Linh Thuy Khanh Tran

**Affiliations:** a Department of Psychology, Grinnell College, USA; b School of Nursing and Medical Technology, University of Medicine and Pharmacy at Ho Chi Minh City, Ho Chi Minh City, Vietnam; c Faculty of Nursing and Medical Technology, Pham Ngoc Thach University of Medicine, Ho Chi Minh City, Vietnam; d Faculty of Nursing, Hong Bang International University, Ho Chi Minh City, Vietnam

**Keywords:** Nursing students, Self-esteem, Parental attachment, Peer attachment, Stress in nursing education, Psychological well-being

## Abstract

**Objectives:**

Managing stress is crucial for the success and well-being of undergraduate nursing students, yet little is known about how interpersonal relationships influence their stress levels. This study explores the impact of parent and peer attachments on stress, focusing on the mediating role of self-esteem. Understanding these dynamics can help educators and counselors develop targeted strategies to support students’ mental health and academic performance.

**Methods:**

A cross-sectional survey was conducted from August to November 2023 at three medical institutions in Ho Chi Minh City, Vietnam. Using a structured self-reported questionnaire, we collected data from 612 nursing students on sociodemographic characteristics, attachments to parents and peers (Inventory of Parent and Peer Attachment), self-esteem (Rosenberg Self-Esteem Scale), and stress levels (Student Nurse Stress Index). Descriptive statistics, correlation analyses, and mediation modeling were employed to examine relationships between these factors.

**Results:**

Pearson’s correlation analyses showed significant associations between parental attachment, peer attachment, self-esteem, and stress, with stress negatively correlated with parental attachment (*r* = -0.42, *p* < 0.001), peer attachment (*r* = -0.38, *p* < 0.001), and self-esteem (*r* = -0.56, *p* < 0.001). Mediation analysis indicated that self-esteem fully mediated the relationship between parental attachment and stress (Sobel test: *Z* = -2.36, *p* < 0.05) and partially mediated the relationship between peer attachment and stress (Sobel test: *Z* = -3.41, *p* < 0.001).

**Conclusions:**

Counselors and school officials could promote parental attachment, peer attachment, and self-esteem in nursing students to reduce stress and improve students’ psychological well-being during their training. Educators should also identify students with insecure attachments to parents or peers to provide timely and appropriate interventions.

Fostering strong parent and peer attachments, along with promoting self-esteem, can serve as a protective factor against stress in nursing students. Educational institutions should implement interventions that enhance supportive relationships and self-worth among students. Identifying those with insecure attachments early on can enable timely psychological support, ultimately improving well-being and academic success in nursing education.

**What does this paper add?**

**What is known?**

• Nursing students experience high levels of stress, which can negatively impact their academic performance, mental health, and career commitment.
• Self-esteem is a key psychological factor influencing stress resilience, but its role in nursing education has been understudied.
• Parental and peer attachments are crucial for psychological well-being, but most existing research focuses on adolescent populations rather than nursing students in higher education.

**What is new?**

• This study is the first to examine the combined effects of parental attachment, peer attachment, self-esteem, and stress among Vietnamese nursing students.
• Self-esteem was found to fully mediate the relationship between parental attachment and stress, suggesting that strong parental bonds help students develop self-worth, lowering stress levels.
• Peer attachment had both direct and indirect effects on stress, indicating that social relationships with peers are a protective factor beyond their influence on self-esteem.
• The findings highlight cultural nuances, particularly in Vietnam’s interdependent society, where family and peer relationships play essential roles in students’ well-being.
• The study provides practical implications for nursing education, emphasizing the need for interventions that strengthen self-esteem, promote healthy peer connections, and support students with insecure attachments.

Alt-text: Unlabelled box dummy alt text

## 1 Introduction

Nursing is a demanding profession that requires both theoretical expertise and hands-on clinical skills. As a result, nursing students experience significant stress as they navigate the challenges of applying knowledge in real-world patient care settings (Ewertsson et al., 2017). A systematic review by Zheng et al. (2022) found that over 61 % of nursing students experience stress due to the pressure of bridging theoretical learning with clinical application, adapting to unfamiliar environments, and managing interpersonal interactions with supervisors, patients, and families. This stress intensifies during internship periods, where students face career uncertainties, shifting learning environments, and high expectations. If left unaddressed, prolonged stress can lead to serious mental and physical health issues, ultimately affecting their career commitment and professional performance (Zheng et al., 2022).

In Vietnam, stress levels among medical students are alarmingly high, with 63.65 % at Hanoi Medical University (Vu and Pham, 2019) and 71.4 % at Ho Chi Minh City University of Medicine and Pharmacy (Tran, 2012) reporting significant stress. Moreover, 52.8 % of students at Ho Chi Minh City University of Medicine and Pharmacy experience combined stress-anxiety-depression disorders (Tran, 2012). Studies in Vietnamese nursing education remain limited, but research at Hai Phong Medical College found a 47.3 % stress rate, with academic pressures, financial burdens, and socioeconomic disparities being key stressors (Ngoc and Tuan, 2023). Many students struggle with tuition fees and must work part-time to afford their education, further exacerbating stress levels (Ngoc and Tuan, 2023).

Given the critical need to address this issue, this study is the first in Vietnam to explore the interplay between interpersonal (parent and peer attachments) and intrapersonal (self-esteem) factors in nursing student stress. By identifying these influences, we aim to provide actionable insights for improving students' mental well-being, academic performance, and overall quality of life. This research is not just timely but essential for shaping a healthier and more resilient nursing workforce.

### 1.1 The role of self-esteem in nursing students

Self-esteem is a critical intrapersonal factor influencing students' academic and psychological well-being, particularly in high-stress disciplines such as nursing (Joseph et al., 2021; Yildirim et al., 2017). Self-esteem is defined as an individual's self-evaluation of their abilities and worth, shaping their confidence, self-satisfaction, and resilience (Chung et al., 2014). Studies have consistently demonstrated that higher self-esteem is associated with academic success, life satisfaction, and reduced engagement in maladaptive behaviors such as poor dietary habits and substance use (Di Giunta et al., 2013; Butkovic et al., 2020; Arsandaux et al., 2020). Furthermore, individuals with high self-esteem exhibit better adaptation to new learning environments, including college, facilitating their ability to manage academic challenges effectively (Butkovic et al., 2020).

In the nursing profession, self-esteem is directly linked to the quality of patient care (Arthur and Randle, 2007), professional communication, empathy, and overall job performance (Dimitriadou-Panteka et al., 2014). Research in South Korea found that self-esteem is pivotal in developing professional identity among nursing students (Min et al., 2021), affecting their commitment to the field, particularly during crises like the COVID-19 pandemic (Nie et al., 2021). Given its far-reaching implications, identifying the factors influencing nursing students' self-esteem is essential in fostering psychological resilience and professional competence.

### 1.2 Parental and peer attachments as key interpersonal factors

Among the many predictors of self-esteem, attachment relationships with parents and peers play a significant role, as these are the primary social interactions students rely on throughout college. Early attachment experiences with parents shape an individual's social expectations and ability to seek support (Delgado et al., 2022). Transitioning to adolescence, individuals progressively form new social support networks, and peer attachments become prominent for their development (Delgado et al., 2022). With that being said, secure parental attachments are a foundation for forming meaningful peer relationships, which later become central sources of intimacy and emotional sharing (Delgado et al., 2022).

Despite spending less time with parents as they grow older, parental attachment is crucial to self-esteem. Research suggests that insecure parental attachment mediates the relationship between parenting practices and self-perception, reinforcing the long-term impact of early bonds (Laible et al., 2004). Significantly, peer support does not fully compensate for the absence of parental support, underscoring the irreplaceable role of parental attachment in shaping self-confidence (Gamble and Roberts, 2005). Nursing students frequently report that parental encouragement and guidance contribute significantly to their self-confidence, highlighting the lasting psychological impact of strong family relationships.

### 1.3 *Attachment and self-esteem: a crucial relationship*

While extensive research has examined the link between parental and peer attachments and self-esteem in adolescents, fewer studies have explored this relationship in nursing students or within the Vietnamese context. However, prior studies indicate that secure attachment relationships with parents and peers strongly predict higher self-esteem (Laible et al., 2004; Fass and Tubman, 2002). Individuals with secure attachments tend to view themselves more positively, benefiting from the consistent emotional support provided by their social networks (Gorrese and Ruggieri, 2013). This positive self-perception helps maintain a stable self-esteem level, fostering resilience in the face of academic and personal challenges.

Conversely, insecure attachment has been linked to low self-esteem and dysfunctional attitudes, leading to later internalizing symptoms such as depression and anxiety (Lee and Hankin, 2009). Studies have found that individuals with insecure attachments struggle with adapting to new situations, experience higher levels of loneliness, and exhibit lower self-esteem, particularly during significant life transitions such as adjusting to college (Kurland and Siegel, 2013). Consequently, nursing students with secure attachments to parents and peers are expected to have higher self-esteem, which may serve as a buffer against academic and professional stressors.

### 1.4 The relationship between self-esteem and stress in nursing students

Research highlights that nursing students face high academic stress levels and often exhibit low self-esteem (Acharya Pandey and Chalise, 2015; Shrestha and Ghimire, 2019). This has significant implications for the nursing profession, as low self-esteem is associated with communication difficulties, reduced work efficacy, and diminished empathy. In contrast, higher self-esteem is linked to better job performance and effective collaboration with patients and colleagues (Dimitriadou-Panteka et al., 2014).

Although no studies have directly explored the relationship between self-esteem and stress among Vietnamese nursing students, research in other countries provides valuable insights. A study in Nepal found a strong negative correlation (*r* = −0.58) between self-esteem and academic stress, indicating that students with higher self-esteem reported significantly lower stress levels (Acharya Pandey and Chalise, 2015). Similarly, a longitudinal study in the UK revealed that nursing students with higher self-esteem experienced lower stress levels throughout their academic journey (Edwards et al., 2010). However, some studies have not found a significant direct relationship between self-esteem and stress (Kataria, 2016; Polk, 2006), suggesting that additional factors may mediate this association.

Despite these mixed findings, self-esteem is widely recognized as a protective factor against stress (Mruk, 2006), as individuals with high self-esteem tend to perceive their abilities more optimistically and manage difficulties with greater resilience. In contrast, students with low self-esteem often struggle to recognize their strengths, experience self-doubt, and are more vulnerable to the adverse effects of stress. Given these findings, fostering self-confidence in nursing students could enhance their psychological resilience, improve their academic performance, and ultimately promote their professional development.

### 1.5 Study rationale and hypotheses

This study seeks to clarify the unique contributions of parental and peer attachments to the psychological well-being of nursing students, focusing on their impact on self-esteem and stress levels. While previous research has established that self-esteem mediates the relationship between attachment and psychological health in adolescence and early adulthood (Wilkinson, 2004), no study has yet examined these variables collectively in Vietnamese nursing students. Addressing this gap is crucial for developing effective strategies to support student well-being and academic success.

Based on existing literature, this study proposes the following hypotheses:

(i) Significant pairwise correlations exist among parental attachment, peer attachment, self-esteem, and stress in Vietnamese nursing students.
(ii) Both parental and peer attachments act as protective factors against stress, meaning that more secure attachments predict lower stress levels.
(iii) Self-esteem mediates the relationships between attachment and stress. Higher attachment security leads to greater self-esteem, which, in turn, reduces stress levels.

**Fig. 1:**
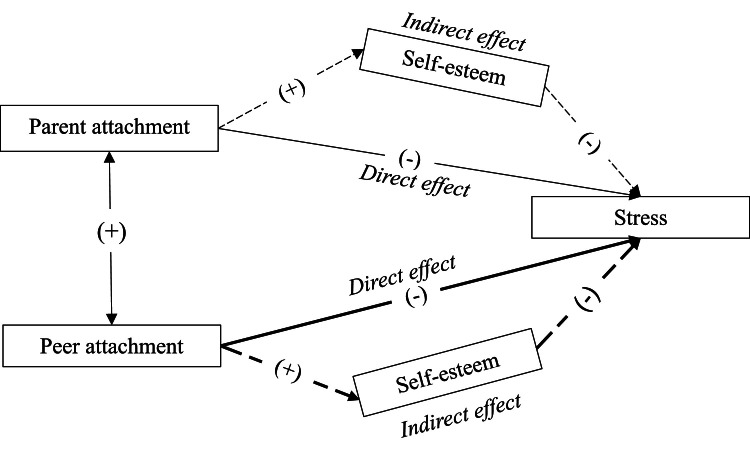
→ Proposed model indicates direct effects of attachment on stress; - - - indicates indirect effects of attachment on stress through self-esteem; ↔ indicates bidirectional correlations between parental and peer attachments.

Fig. 1 presents a conceptual model illustrating these pathways. By examining these relationships, this study aims to provide valuable insights for improving nursing education, fostering student resilience, and enhancing psychological well-being.

## 2 Methods

### 2.1 Study design

This study employed a cross-sectional survey design using self-reported questionnaires and a convenience sampling method to assess parental and peer attachments, self-esteem, and stress among nursing students.

### 2.2 Participants and setting

A convenience sample of undergraduate nursing students was recruited from August 2023 to November 2023 across three medical universities in Ho Chi Minh City, Vietnam: University of Medicine and Pharmacy at Ho Chi Minh City, Pham Ngoc Thach University of Medicine, and Hong Bang International University. Participants were evenly distributed across the three institutions. The study was approved by the Ethics Committee of University of Medicine and Pharmacy at Ho Chi Minh City (IRB-VN01002/IORG0008603/FWA00023448). Before participating, all students were provided with written informed consent forms, which they were required to sign and submit along with their completed surveys. To maintain confidentiality, no personal identifying information was collected.

### 2.3 Study instruments

The survey collected demographic and background information, including the participant's university affiliation, gender, date of birth, class year, and living situation. Additionally, three validated instruments assessed attachment relationships (Inventory of Parental and Peer Attachment – IPPA (Armsden and Greenberg, 1987)), self-esteem (Rosenberg Self-Esteem Scale – RSE (Rosenberg, 1965)), and perceived stress (Student Nurse Stress Index – SNSI (Jones and Johnston, 1999)).

All instruments underwent forward-backward translation by bilingual experts to ensure linguistic and conceptual equivalence between English and Vietnamese versions. Then, content validity of the translated instruments was assessed by six experts using the Content Validity Index (CVI) to evaluate item relevance and clarity (Engelbrecht, 2022). Overall, the three instruments were judged as having excellent content validity, with I-CVI = 1.00 and S-CVI/UA = 1.00 across all items, except for item 6 (I-CVI = 0.83) in self-esteem scale (S-CVI/UA = 0.90). This item was subsequently revised to improve its relevance and clarity.

#### 2.3.1 Inventory of Parental and Peer Attachment

The Inventory of Parental and Peer Attachment (Armsden and Greenberg, 1987) was used to assess attachment relationships with parents and peers across three dimensions: Trust, Communication, and Alienation (reverse-scored). The IPPA consists of 28 items measuring parental attachment and 25 items measuring peer attachment, all scored on a 5-point Likert scale (1 = Strongly disagree, 5 = Strongly agree). Higher total scores indicate stronger attachment relationships with parents or peers. The instrument has demonstrated good psychometric properties, with validation studies confirming adequate internal consistency (Cronbach's alpha ranging from 0.72 to 0.91) and stable factor structure in adolescent populations (Maya et al., 2023; Habibi Asgarabad et al., 2025).

#### 2.3.2 Rosenberg Self-Esteem Scale

The Rosenberg Self-Esteem Scale (Rosenberg, 1965), a widely used 10-item measure, assessed global self-esteem by evaluating positive and negative self-perceptions. Responses were rated on a 4-point Likert scale (1 = Strongly disagree, 4 = Strongly agree). Items assessing negative self-perceptions were reverse scored, with higher total scores indicating higher self-esteem. The RSE has demonstrated strong psychometric properties across diverse populations, with validation studies reporting good internal consistency (Cronbach's alpha = 0.77 to 0.88) and satisfactory construct validity (Jelisavac, 2024; Tinakon and Nahathai, 2012).

#### 2.3.3 Student Nurse Stress Index

Perceived stress among nursing students was measured using the 22-item Student Nurse Stress Index (Jones and Johnston, 1999), which assesses four domains of nursing student stress: Academic load, Clinical concerns, Personal problems, and Interface worries (stress related to balancing academic and personal life). Responses were recorded on a 5-point Likert scale (1 = Not stressful at all, 5 = Extremely stressful). Higher total scores indicate greater levels of perceived stress. This instrument has shown adequate reliability and validity in nursing student populations, with studies reporting good internal consistency (Cronbach's alpha > 0.80) and confirmed factor structure (Ding et al., 2023; Engelbrecht, 2022).

### 2.4 Procedure

Data collection took place in randomly selected lecture sessions. Physical copies of the survey and consent forms were distributed to students during breaks or at the end of lectures. Participants were instructed to complete and return the surveys on the same day. Participation was voluntary and uncompensated, and confidentiality was ensured throughout the study. Completed physical surveys were scanned and digitized for further analysis.

### 2.5 Data analysis

Data analysis was conducted using R Version 4.4.1 (Posit team, 2022). Key steps included:

Data Standardization: Numeric responses for the main study variables were standardized and averaged within each scale to ensure instrument consistency.
Correlation Analysis: Pearson’s correlation tests examined bidirectional relationships between parental attachment, peer attachment, self-esteem, and stress.
Mediation Analysis: A three-step regression approach (Baron and Kenny, 1986) assessed whether self-esteem mediates the relationship between parental/peer attachment and stress.
Sobel Test: The Sobel test (Sobel, 1986) was conducted to determine the statistical significance of the indirect mediation effects.

All statistical tests were performed with a 95 % confidence level, and p-values < 0.05 were considered statistically significant.

## 3 Results

### 3.1 Sociodemographic characteristics

A total of 612 responses were collected, of which 384 (62.7 %) were deemed valid after excluding responses that failed both attention check questions or had missing data exceeding 10 % of any measure. Table 1 provides a summary of the descriptive statistics for the final sample. Participants were drawn from three universities, with 119 students (31.0 %) from University of Medicine and Pharmacy at Ho Chi Minh City, 126 students (32.8 %) from Pham Ngoc Thach University of Medicine, and 139 students (36.2 %) from Hong Bang International University.

**Table 1:** Descriptive statistics of participants.

	N	%
**Gender**		
Female	289	75.26
Male	89	23.18
NA	6	1.56
**Class year**		
Freshmen	131	34.11
Sophomore	107	27.86
Junior	68	17.71
Senior	77	20.10
NA	1	0.26
**Living situation**		
Home with parents	199	51.82
Rents with friends	103	26.82
Rent alone	28	7.29
Dorm	18	4.69
Others	32	8.33
NA	4	1.04
**Year of birth**		
1987	1	0.26
1990	1	0.26
1995	1	0.26
1997	1	0.26
2000	4	1.04
2001	5	1.30
2002	73	19.01
2003	68	17.71
2004	99	25.78
2005	111	28.91
NA	20	5.21

N: Number of students within sub-group.

NA: Not Available.

Regarding academic standing, the sample included 131 first-year students (34.1 %), 107 second-year students (27.9 %), 68 third-year students (17.7 %), and 77 fourth-year students (20.1 %). One participant (0.3 %) did not specify their class year. Most respondents were female (*n* = 289, 75.3 %), while 89 participants (23.2 %) identified as male, and six participants (1.6 %) did not specify their gender. In terms of living arrangements, 199 students (51.8 %) lived with their parents, 103 students (26.8 %) rented accommodation with friends, 28 students (7.3 %) lived alone, 18 students (4.7 %) resided in dormitories, and 32 students (8.3 %) reported other living arrangements. Additionally, four participants did not respond to the question regarding their living situation. This distribution provides insight into the demographic diversity of the sample, ensuring that the findings reflect a broad range of experiences among nursing students across different universities and academic levels.

### 3.2 Descriptive statistics and correlation analysis

One-way ANOVAs were conducted to examine potential differences in stress levels based on demographic factors. Results indicated a significant effect of institutional affiliation on perceived stress, *F* (2, 315) = 8.965, *p* < 0.001. Post-hoc comparisons revealed that Pham Ngoc Thach University of Medicine nursing students reported the highest stress levels (*M* = 2.71, SD = 0.26). In contrast, Hong Bang International University students reported the lowest stress levels (*M* = 2.55, SD = 0.30).

Pearson’s correlation analyses examined the relationships between parental attachment, peer attachment, self-esteem, and stress. Table 2 presents the results, demonstrating that all four variables were significantly correlated. Specifically, parental and peer attachments were positively correlated, suggesting that students with strong relationships with their parents also tend to develop secure peer relationships. Both parental and peer attachments were positively associated with self-esteem, indicating that students with stronger attachment bonds tend to have higher self-esteem. Additionally, stress was negatively correlated with parental attachment, peer attachment, and self-esteem, suggesting that students with higher attachment security and greater self-esteem experienced lower stress levels. These findings support the hypothesis that interpersonal relationships and self-esteem are crucial in mitigating stress among nursing students.

**Table 2:** Correlations between attachments, self-esteem, and stress.

	M	SD	1	2	3
1. Parental attachment	3.63	0.98			
2. Peer attachment	3.50	0.95	0.35***		
3. Self-esteem	2.79	0.68	0.35***	0.48***	
4. Stress	2.71	1.02	−0.13**	−0.29***	−0.27***

*p < 0.05 **p < 0.01 ***p < 0.001.

### 3.3 Mediation analyses

#### 3.3.1 The mediating role of self-esteem in the relationship between parental attachment and stress

A series of three regression analyses were conducted to examine whether self-esteem mediates the relationship between parental attachment and stress. The results are summarized in Table 3. Equation (1) assessed the direct effect of parental attachment on stress, equation (2) evaluated the direct effect of parental attachment on self-esteem, and equation (3) examined the combined effects of parental attachment and self-esteem on stress. Results from simple linear regression analyses indicated that parental attachment significantly predicted stress levels (*B* = 3.83, *p* < 0.001) as well as self-esteem (*B* = −0.36, *p* < 0.001). In the final regression model, parental attachment and self-esteem remained significant predictors of stress, suggesting that self-esteem plays a mediating role in this relationship.

**Table 3:** Summary of regression with parental attachment as the predictor of stress.

Predictor Variable	Equation (1)			Equation (2)			Equation (3)		
	B	SE	*p*	B	SE	*p*	B	SE	*p*
Intercept	3.83	0.22	<0.001***	1.47	0.13	<0.001***	4.16	0.25	<0.001***
Parental attachment	−0.30	0.05	<0.001***	−0.36	0.04	<0.001***	−0.23	0.07	<0.001***
Self-esteem							−0.21	0.09	0.015*

Equation (1) - parental attachment as the predictor and stress as the outcome. Equation (2) - parental attachment as the predictor and self-esteem as the outcome. Equation (3) - parental attachment and self-esteem as predictors and stress as the outcome. *p < 0.05 **p < 0.01 ***p < 0.001.

A Sobel test was conducted to assess the significance of this mediation effect further, confirming that self-esteem significantly mediated the relationship between parental attachment and stress (*Z* = −2.36, *p* < 0.05). These findings suggest that self-esteem fully mediates the impact of parental attachment on stress, indicating that students with strong parental attachment experience lower stress levels primarily due to enhanced self-esteem. This highlights the crucial role of self-esteem as a psychological buffer in reducing stress among nursing students.

#### 3.3.2 The mediating role of self-esteem in the relationship between peer attachment and stress

A series of three regression analyses were performed to investigate whether self-esteem mediates the relationship between peer attachment and stress. The results are summarized in Table 4. Equation (a) evaluated the direct effect of peer attachment on stress, equation (b) assessed the direct effect of peer attachment on self-esteem, and equation (c) examined the combined effects of peer attachment and self-esteem on stress. The regression analyses revealed that peer attachment significantly predicted stress (*B* = 3.33, *p* < 0.05) and self-esteem (*B* = 0.32, *p* < 0.001). However, when both peer attachment and self-esteem were included in the final model, peer attachment (*B* = −0.37, *p* < 0.001) remained a significant predictor of stress, whereas self-esteem did not (*B* = 0.08, *p* = 0.587). A Sobel test further confirmed that self-esteem significantly mediated the relationship between peer attachment and stress (*Z* = −3.41, *p* < 0.001), supporting a partial mediation effect. These findings indicate that peer attachment reduces stress levels in nursing students, in part through its positive influence on self-esteem. However, unlike parental attachment, which was fully mediated by self-esteem, peer attachment directly affects stress, suggesting that social connections with peers independently contribute to stress reduction beyond the influence of self-esteem.

**Table 4:** Summary of regression with peer attachment as the predictor of stress.

Predictor Variable	Equation (a)			Equation (b)			Equation (c)		
	B	SE	*p*	B	SE	*p*	B	SE	*p*
Intercept	3.33	0.29	<0.001***	1.65	0.18	<0.001***	3.92	0.32	<0.001***
Peer attachment	−0.17	0.08	0.03*	0.32	0.05	<0.001***	−0.04	0.08	0.587
Self-esteem							−0.37	0.09	<0.001***

Equation (1) - peer attachment as the predictor and stress as the outcome. Equation (2) - peer attachment as the predictor and self-esteem as the outcome. Equation (3) - peer attachment and self-esteem as predictors and stress as the outcome. *p < 0.05 **p < 0.01 ***p < 0.001.

## 4 Discussion

This study examined the distinct roles of parental and peer attachment in stress among Vietnamese nursing students and the mediating effect of self-esteem in these relationships. Consistent with our hypotheses, significant pairwise correlations were observed between parental attachment, peer attachment, self-esteem, and stress, reinforcing previous research that suggests secure parental attachments are associated with stronger peer relationships (Fass and Tubman, 2002; Armsden and Greenberg, 1987). High-quality parental attachment provides a secure base for emotional support and shapes expectations for future social interactions (Delgado et al., 2022), which may explain why students with strong parental bonds also report higher-quality peer attachments.

Moreover, both parental and peer attachments were found to be strong predictors of self-esteem. In Vietnam, where familial bonds and community ties remain strong even during college years (Mai and Le, 2023), many students continue to live with their parents, maintaining emotional closeness and receiving ongoing support (Karunarathne, 2023). This suggests that Vietnamese college students may simultaneously benefit from parental and peer attachments, creating a robust support system fostering self-worth and psychological stability. As a result, secure attachments to parents and peers help students develop positive self-perceptions, leading to higher self-esteem (Karunarathne, 2023).

### 4.1 The protectiveroleof attachment against stress

This study's findings confirm that parental and peer attachments serve as protective factors against stress in nursing students, with self-esteem mediating these relationships. Students with stronger attachments reported lower stress levels, and this effect was significantly mediated by higher self-esteem. Lazarus and Folkman’s transactional model of stress and coping suggested stress as a product of the interaction between an individual and their environment (Lazarus and Folkman, 1984). An event is considered a stressor only after an individual appraises it as threatening or harmful to their well-being, such that the event exceeds their resources for coping. Specifically, person variables (e.g., self-esteem) and environmental variables (e.g., social support network) are the antecedents that influence the appraisal of the stressful event and the subsequent coping strategies (Lazarus, 1990), suggesting that self-esteem and attachment relationships are important factors in one’s appraisal and response to stressors. Secure attachments to parents and peers contribute to the development of a stable and positive self-concept, enhancing students' appraisal of their coping abilities and their perceived access to social support. Therefore, self-esteem not only mediates but also operationalizes part of the coping potential in response to stress.

In particular, self-esteem was found to fully mediate the relationship between parental attachment and stress, indicating that parental support functions primarily through building intrapersonal resources, which fosters a stable self-concept and helps students navigate stress more effectively. This aligns with Vietnam’s cultural emphasis on filial piety, where even in adulthood, parental influence is crucial in self-esteem development (Mai and Le, 2023).

In contrast, the effect of peer attachment on stress was only partially mediated by self-esteem, suggesting that peer relationships directly buffer stress beyond their influence on self-esteem. This finding is particularly relevant in the college context, where students increase their social interactions with peers while seeking greater independence from parents (Fass and Tubman, 2002). Given peers' significant role in navigating academic and clinical stress, peer attachment likely provides direct emotional and practical support, helping students cope with stress independent of self-esteem.

Acknowledging the collectivistic cultural values in Vietnam that help explain the sustained effects of parental attachment on one’s psychological well-being in early adulthood, it is vital to compare these findings to studies from more individualistic cultures to highlight the key cultural distinction. While collectivistic cultures embrace the interdependent relationships between group members, individualistic cultures, such as the US or most Western countries, prioritize individuals’ autonomy, independence, and pursuit of personal goals (Cheng et al., 2020). As college students spend more time with peers than parents – which is particularly true in individualistic cultures, parental attachment in individualistic countries tends to play a less prominent role in young adults’ self-esteem, and peer support becomes more dominant (Wilkinson, 2004). Nonetheless, secure attachments with parents and peers are consistently associated with higher self-esteem cross-culturally (Laible et al., 2004; Fass and Tubman, 2002; Gorrese and Ruggieri, 2013); (Xu and Umemura, 2025). Similarly, a higher sense of social support is linked with less psychological distress, such as depressive symptoms, loneliness, and stress, in both collectivistic and individualistic cultures (Xu and Umemura, 2025; Camara et al., 2014).

While the nature of attachment relations may vary across cultures, the protective role of secure parental and peer attachments in promoting self-esteem and reducing stress appears to be universal. These findings also underscore the complexity of attachment relationships and their interplay with self-esteem and stress. The Vietnamese cultural context further highlights the differential roles of parental and peer attachments, emphasizing the continued importance of parental support while acknowledging the growing role of peer relationships in college students' well-being.

### 4.2 Practical implications for school administrators and counselors

The correlation analyses further emphasize the significant relationship between self-esteem, attachment quality, and stress in Vietnamese nursing students. Given that self-esteem is associated with enhanced academic performance (Weisskirch, 2018), greater academic motivation (Almansour, 2023), and increased resilience to stress (Sharma and Bali, 2013), it is essential that school administrators and counselors implement targeted interventions to promote self-esteem. Programs such as self-affirmation exercises and mindfulness training can encourage positive self-perception (Valiente et al., 2020) while reducing negative self-talk (Mon et al., 2016). These interventions can be integrated into nursing curricula, incorporated into in-class activities, or offered as extracurricular workshops to reinforce self-esteem in students.

Furthermore, according to the present findings, administrators should proactively identify students with weak attachment relationships, as these students are more vulnerable to stress. Attachment-based interventions should be implemented early, as they have been shown to enhance self-esteem and foster positive social relationships in college students with insecure attachment styles (Kilmann et al., 2006). Moreover, interventions should be tailored based on the specific attachment deficit. Since peer attachment was found to be more directly related to stress than parental attachment, students with weaker peer relationships may require peer-support interventions, group-based counseling, or mentorship programs rather than self-esteem-focused strategies. Conversely, self-esteem-enhancing programs may be more effective in reducing stress for students with weaker parental attachments. This differentiation in intervention strategies could lead to more personalized and impactful mental health support for nursing students.

### 4.3 Future research directions

Given that stress is a complex multivariate process (Mai and Le, 2023) and the strong association between parental and peer attachments found in this study, future studies should further explore the parental and peer attachments’ joint influence on self-esteem and stress while considering involving other important variables in the equation. Additionally, research should investigate cross-contextual variations, considering students from diverse demographic and socioeconomic backgrounds. For instance, future studies could focus on historically marginalized student populations, who may experience unique stressors beyond those examined in this study.

To improve methodological rigor, future research should utilize digital survey platforms to reduce data entry errors and consider integrating objective psychological assessments for measuring self-esteem, attachment quality, and stress. Due to the nature of a cross-sectional study, no causal relationships were established in the present study. To address this limitation, future research should also employ longitudinal designs to examine causal pathways between attachment, self-esteem, and stress. Finally, this study's findings provide a foundation for developing culturally tailored interventions that enhance the psychological well-being of nursing students in Vietnam and similar educational contexts.

## 5 Conclusion

This study provides critical insights into the role of attachment relationships and self-esteem in stress management among Vietnamese nursing students. The findings emphasize that secure parental and peer attachments serve as protective factors against stress, with self-esteem playing a key mediating role. While parental attachment reduces stress primarily by fostering self-esteem, peer attachment directly buffers stress beyond its influence on self-esteem. These results highlight the importance of attachment-based and self-esteem-focused interventions in nursing education. By recognizing the distinct yet interconnected influences of family, peer relationships, and self-concept, institutions can develop more effective mental health strategies to enhance student well-being and academic success.

## Source(s) of support

This work was conducted without any external funding or financial support.

## CRediT authorship contribution statement

**Quynh Brooke Ho:** Writing – review & editing, Writing – original draft, Resources, Project administration, Methodology, Investigation, Formal analysis, Data curation, Conceptualization. **Huong Thi Lan Tran:** Writing – review & editing, Methodology, Investigation, Data curation, Conceptualization. **Dung Thi Thuy Nguyen:** Writing – review & editing, Investigation, Data curation, Conceptualization. **Thuan Thi Tran:** Writing – review & editing, Methodology, Investigation, Data curation, Conceptualization. **Linh Thuy Khanh Tran:** Writing – review & editing, Writing – original draft, Validation, Supervision, Resources, Project administration, Methodology, Investigation, Formal analysis, Data curation, Conceptualization.

## Declaration of competing interest

Conflicting Interest (If present, give more details): Nil
